# Guided, Fusion-Based, Large Depth-of-field 3D Imaging Using a Focal Stack

**DOI:** 10.3390/s19224845

**Published:** 2019-11-07

**Authors:** Yuhao Xiao, Guijin Wang, Xiaowei Hu, Chenbo Shi, Long Meng, Huazhong Yang

**Affiliations:** 1 Department of Electronic Engineering, Tsinghua University, Beijing 100084, China; xyh18@mails.tsinghua.edu.cn (Y.X.); hu-xw15@mails.tsinghua.edu.cn (X.H.); yanghz@tsinghua.edu.cn (H.Y.); 2 ShanDong MingJia Technology Co., Ltd., Taian 271021, China; shichenbo@gmail.com (C.S.); mengl@mingjiachina.com (L.M.)

**Keywords:** 3D imaging, focal stack, guided fusion

## Abstract

Three dimensional (3D) imaging technology has been widely used for many applications, such as human–computer interactions, making industrial measurements, and dealing with cultural relics. However, existing active methods often require both large apertures of projector and camera to maximize light throughput, resulting in a shallow working volume in which projector and camera are simultaneously in focus. In this paper, we propose a novel method to extend the working range of the structured light 3D imaging system based on the focal stack. Specifically in the case of large depth variation scenes, we first adopted the gray code method for local, 3D shape measurement with multiple focal distance settings. Then we extracted the texture map of each focus position into a focal stack to generate a global coarse depth map. Under the guidance of the global coarse depth map, the high-quality 3D shape measurement of the overall scene was obtained by local, 3D shape-measurement fusion. To validate the method, we developed a prototype system that can perform high-quality measurements in the depth range of 400 mm with a measurement error of 0.08%.

## 1. Introduction

Three dimensional (3D) imaging based on structured light has been widely used in many applications, such as for human–computer interactions, industrial measurements, and regarding cultural relics [1,2,3]. There are numerous structured-light methods which have been discussed thoroughly by Salvi et al. [4] and Zhang [5]. However, due to the limited light source power and low sensor quantum efficiency, these existing methods often require a large aperture to maximize the light throughput from the light source to the camera, which causes the depth of field (DOF) of the projector and camera to reduce. The common DOF between the projector and camera is the working volume of the structured light 3D imaging system, which is shallow as a result. That fundamental limitation makes it difficult for the system to be used to measure scenes with large depth variation. To make the structured light 3D imaging system work for general real-world scenes, it is essential to extend the working volume of the system, which is defined as the DOF of the system in this paper.

The state-of-the-art in extending DOF methods of the structured light 3D imaging system focus on the projector’s DOF extension mainly, assuming that the camera has an infinite DOF, which will not hold in the case of large aperture requirement. This paper considers the camera’s and projector’s DOF extension simultaneously and presents a novel method to extend DOF of the structured light 3D imaging system using the MEMS mirror-enabled laser projector and the liquid-lens-based imaging system. For projector DOF extension, we replaced the traditional DLP projector with the MEMS mirror-enabled laser projector whose displayed images are in focus everywhere [6]. The optics of these projectors are fundamentally different from DLP projectors, which use laser light sources and do not suffer from focusing issues. For camera DOF extending, we adopted the liquid-lens-based imaging system to capture images to form a focal stack, which was able to generate the coarse depth map of the scene to guide local 3D measurement fusion. It is vital for 3D measurement systems to know for certain, the focal length, and keep it unchanged during calibration. Thus, extending the working range of 3D imaging systems using a liquid lens can achieve a highly stable optical performance. Specifically, we (1) captured structured light patterns with different focal settings to measure objects in the scene; (2) extracted a texture map from each focus position into the focal stack to generate the global coarse depth map; (3) fused each local measurement under the guidance of the global coarse depth map to generate the high-quality 3D measurement covering the overall scene. Our prototype system can work effectively for the depth range of 200–600 mm with the measurement error of 0.08%.

The rest of our paper is organized as follows. In Section 2, we review prior approaches that are related to our proposed method. Then, we explain the principle of the method we propose in Section 3. In Section 4, experiments on real data are presented to verify the effectiveness of the method. In Section 5, we discuss the limitations of the method. Finally, we summarize the paper in Section 6.

## 2. Related Work

In this section, we first review the state-of-the-art large DOF methods of the structured light 3D imaging system. Then, we introduce camera DOF extension studies, which are related to our method.

### 2.1. DOF Extension of the Structured Light 3D Imaging System

For the DOF extension of the structured light 3D imaging system, the main work is focused on the projector, assuming that the camera has an infinite DOF. Zhang and Nayar exploited the effect of projector defocusing as a cue to recover 3D shape in [7]. They developed a novel, temporal, defocus analysis method to recover depth at each camera pixel by estimating the projected pattern blur occurring in the frequency domain. They could recover accurate depth maps, but the effects of global light transport could not be handled well. Gupta et al. presented a method to model both projector defocus and global illumination simultaneously [8]. They illustrated that both these seemingly disparate effects could be expressed as low pass filters on the incident illumination. Gupta and Nayar proposed projecting sinusoidal patterns with frequencies limited to a narrow, high-frequency band. These patterns produce a set of images over which defocus’s effects remain constant for each point in the scene. Thus, the projector lens’s defocusing effect can be attenuated as much as possible [9]. However, this method’s accuracy relies on the user’s selection of the pattern frequency. Moreover, the information about the pattern degrades rapidly when it is faced with severe defocus; thus, it can only extend the working range to some extent. In [10], illumination defocus is exploited in a different way, which generates sinusoidal patterns by projecting binary patterns with a defocused projector. Thus, the phase shift algorithm could run in real-time and recover dynamic scenes. Achar and Narasimhan extended the working volume by projecting structured light patterns with different focus settings [11]. However, this approach is volatile due to the mechanically tunable lens. The seminal works of Brown et al. [12] were the first to improve projection quality by compensating defocus blurs using image pre-compensation. They estimated a series of spatially-varying point-spread-functions across the projector’s image, which were then used to guide preprocessing based on Wiener filtering to condition the image before projection. This technique works well for small kernels but is less effective on large kernels. Zhang et al. proposed focal length and exposure adaption in [13]. They changed the illumination pattern and intensity locally based on the prior depth information. However, the accuracy of depth values measured by this method is not high. A light field projector was introduced by Kawasaki et al. [14], which is realized by attaching a coded aperture with a high-frequency mask. However, the coded aperture attenuated the light throughout and resulted in a decrease in the signal-to-noise ratio.

### 2.2. DOF Extension of the Camera

For the DOF extension of the camera, Kuthirummal et al. presented a novel imaging system which varies the position of the image detector during the integration time of a single photograph to extend DOF [15]. However, this system is highly dependent on the hardware device, and so it is rather complicated and power-consuming. Levin et al. proposed a modification to the conventional camera, which inserts a patterned occlusion within the aperture of the camera lens to recover all-in-focus images [16]. However, the applicable scenarios for that modification are limited, and depth recovery is not robust. Wavefront coding is a promising approach to extend DOF imaging [17,18]. An all-in-focus image can be obtained by deconvolving the captured image with a single blur kernel. Georgeiv et al. developed a prototype integral camera which samples the 4D light field of a scene to compute all in-focus images [19]. However, this camera is limited by a fundamental trade-off between spatial resolution and angular resolution. A related approach is to capture many images to form a focal stack [20,21]. This method can generate an all-in-focus image of the scene with a depth map effectively. To reduce the capture time of a focal stack, Hasinoff and Kutulakos proposed a technique which uses a combination of different apertures and focal plane locations [22].

The aforementioned camera’s DOF extension methods cannot be directly employed for large depth-range 3D shape measurements, since they are primarily designed for getting focused 2D images without exactly knowing the altered physical parameters (e.g., focal length), while for any DOF extending methods to be adopted in 3D shape measurement, any changes of a physical parameter have to be precisely known. Fortunately, the focus stacking technique can compute a coarse depth map of the scene, which could be exploited to enlarge the working range of structured light 3D imaging systems effectively. The detailed content will be discussed in the following sections.

## 3. Computational Framework for Large DOF 3D Imaging

**Figure 1:**
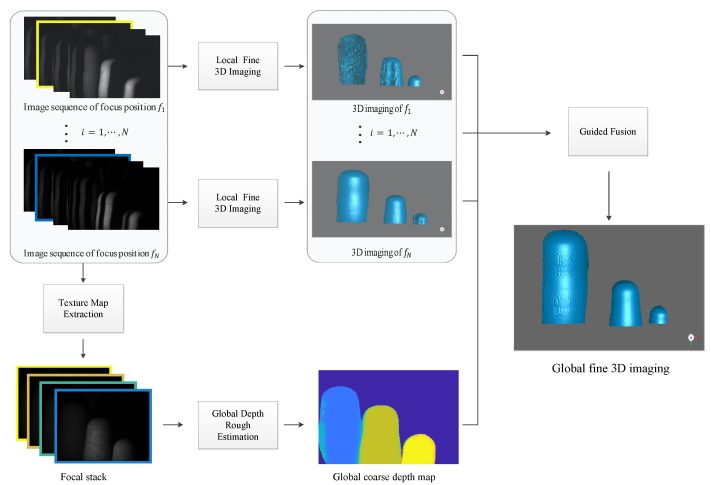
The framework of our proposed method. Image sequences of different focal distances are acquired by the multi-focus illumination-imaging system; then, local, fine 3D measurements and a global coarse depth map are generated by the gray-code method and focus stacking technique, respectively. These local, fine 3D measurements, along with the global coarse depth map, are finally fed into the guided fusion model to get the high-quality 3D measurement of the overall scene.

The framework of the method we propose is shown in Figure 1. The overall process could be divided into four modules, which are local fine 3D Imaging, texture map extraction, global depth rough estimation, and guided fusion. First, we project the structured light patterns over the scene through the MEMS mirror-enabled laser projector and then use the imaging system equipped with a liquid lens to capture a sequence of images focusing at various depth of the scene. At each focus position, we adopt the “Local Fine 3D Imaging” module to measure 3D shape of the object and extract a texture map through the “Texture Map Extraction” module. The texture maps are then combined into the focal stack to generate the global coarse depth map with the module of “Global Depth Rough Estimation.” Finally, the high-quality measurement of the overall scene, which is composed of the accurate part of local measurements, is obtained through the “Guided Fusion” module. The principle of each module will be discussed in subsections that follow.

### 3.1. Local Depth Fine Estimation and Texture Map Extraction

The structured light technique is one of the extensively adopted methods to recover depth information of the scene. A projection device is utilized to project structured patterns over the object actively. The projected, structured patterns carry encoded information to establish the correspondence between the camera and the projector. Once the system is calibrated, 3D coordinates of the scene can be generated well, based on the ray-plane triangulation. In this paper, we choose the gray code method as the local 3D measurement due to the benefits of being easy to operate and producing accurate reconstructions of objects which are within the working range [23].

**Figure 2:**
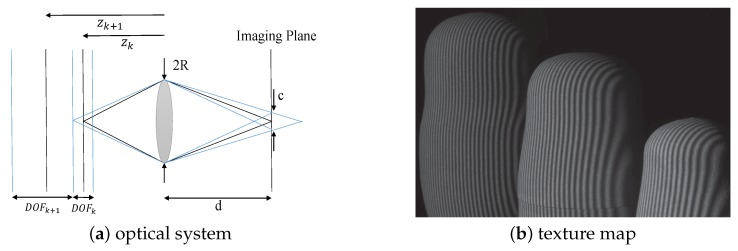
Optimal focus positions and texture map extraction. (**a**): optical system; (**b**): texture map. The optimal sequence of focus positions has no gaps or overlaps between depths of field (DOFs) from consecutive focal planes. The image projected by the finest stripes of the gray code sequence is extracted into the focal stack.

For generating a focal stack, the optimal sequence of focus positions is obtained based on [24]. Specifically, let us consider the optical system focused at distance $z_{k}$ from the lens, as shown in Figure 2a. There is a depth range $DOF_{k}$ around $z_{k}$ for which the scene is in-focus in the imaging plane. Based on the thin lens model and geometrical considerations, the near limit, $z_{k}^{\left(1\right)}$, and far limit $z_{k}^{\left(2\right)}$ of $DOF_{k}$, satisfy:

(1) $$\frac{1}{z_{k}^{\left(1\right)}}-\frac{1}{z_{k}}=\frac{c}{2Rd}$$

(2) $$\frac{1}{z_{k}}-\frac{1}{z_{k}^{\left(2\right)}}=\frac{c}{2Rd},$$

where *R* is the radius of the aperture, *d* is the fixed distance between the imaging plane and the lens, and *c* is the diameter of the circle of confusion. The condition to obtain an optimal sequence of focus positions is that the far limit of $DOF_{k}$ coincides exactly with the near limit of $DOF_{k+1}$ of the next focal distance $z_{k+1}$:

(3) $$z_{k}^{\left(2\right)}=z_{k+1}^{\left(1\right)},$$

and then, a recursion formula, given the foreground distance (k=1) $z_{1}$, is derived to compute the optimal set of focal distance:

(4) $$z_{k+1}={\left(\frac{1}{z_{k}}-\frac{c}{Rd}\right)}^{-1}.$$

After determining the focus positions, we need to pick a texture map from the captured images at each focus position to form the focal stack. The sharpness of the texture change is important for the focus stacking technique to distinguish which focus position is in-focus. Since there may exist texture-less objects in the general scene, we need to add texture to the surface of the objects. The gray coded patterns vary from coarse stripes to fine stripes gradually. Therefore, we regard the structured light projected on the surface of the object as its "surface texture," and extract the image which is projected by the finest stripe from each focus position to combine the focal stack, as shown in Figure 2b.

### 3.2. Global Depth Rough Estimation

The focus stacking technique is a computational approach to extend the camera DOF effectively. However, the all-in-focus image cannot be directly employed for the reason that we cannot know the altered physical parameters (e.g., focal length) exactly. Thanks to the by-product of the focus stacking technique, we could get the coarse depth map of the scene, which can guide local 3D measurements to fuse into the global 3D measurement with high quality.

As discussed before, we extract texture maps from various focus distances and combine them into the focal stack. Then, given the magnitude of gradient as the sharpness measure, we calculate the gradients of all the images in the stack by

(5) $$G_{i}\left(x,y\right)=\left|\nabla I_{i}\left(x,y\right)\right|=\sqrt{{\left(\frac{\partial I_{i}}{\partial x}\right)}^{2}+{\left(\frac{\partial I_{i}}{\partial y}\right)}^{2}},$$

where $G_{i}\left(x,y\right)$ is the gradient of $I_{i}\left(x,y\right)$ which is the ith image in the focal stack. After that, the global coarse depth map can be obtained by

(6) $$D\left(x,y\right)=\underset{i}{\mathrm{argmax}}G_{i}\left(x,y\right),$$

where D(x,y) stores the index of image that has the maximum gradient of each pixel in the focal stack. This depth map is also known as a focus map. However, due to the large blur kernel case, the edges will spread on a large scale. Thus, many wrong values in the depth map may result. In our previous work [20], we proposed a max-gradient flow (MGF) method to remove false edges and formulated an anchored, rolling-filtering approach to refine the depth map, which effectively reduces the artifacts caused by large blur scale. Here, the main steps in MGF will be reviewed as follows:

First, we define the 2D max-gradient flow as

(7) $$MGF\left(x,y\right)={\left[f_{x}\left(x,y\right),f_{y}\left(x,y\right)\right]}^{T},$$

where

(8) $$\left[\begin{array}{c} f_{x}\left(x,y\right)\\ f_{y}\left(x,y\right) \end{array}\right]=\left[\begin{array}{c} \frac{\max_{j}G_{j}\left(x+\Delta x,y\right)-\max_{i}G_{i}\left(x,y\right)}{\Delta x}\\ \frac{\max_{k}G_{k}\left(x,y+\Delta y\right)-\max_{i}G_{i}\left(x,y\right)}{\Delta y} \end{array}\right]$$

The flow describes the change of maximum gradient along each axis (i.e., the propagation of gradient in the stack). And the direction of propagation can be easily derived as

(9) $$\theta \left(x,y\right)=arctan\left(\frac{f_{y}\left(x,y\right)}{f_{x}\left(x,y\right)}\right).$$

Then, with the max-gradient flow, we can extract source point (x,y) if

(10) $$f_{x}\left(x+\delta x,y\right)>0\;and\;f_{x}\left(x-\delta x,y\right)<0,$$

which means that it is the source where the max-gradient starts propagating. All the source points form a sparse set, which represents the true edges in the scene. Finally, we formulate an anchored rolling filtering approach to refine the depth map with the extracted true edges. Since the depth map and the all-in-focus image are closely related, we form a ping-pong approach that estimates them in turn. Starting from the initial depth map, the all-in-focus image and the depth map are updated iteratively in a rolling way. Suppose that the depth map *D* is given, the all-in-focus image FI can be composited by extracting the pixel from the stack:

(11) $$FI\left(x,y\right)=I_{D(x,y)}\left(x,y\right).$$

And suppose that the all-in-focus image FI is available, the depth map *D* can be refined by a joint bilateral filtering approach [25]:

(12) $$\hat{D}\left(p\right)=\frac{1}{K_{p}}\sum_{p^{\prime }\in N_{p}}exp\left(-\frac{{∥p^{\prime }-p∥}^{2}}{2{\sigma_{s}}^{2}}-\frac{{∥FI\left(p^{\prime }\right)-FI\left(p\right)∥}^{2}}{2\sigma^{2}}\right)D\left(p^{\prime }\right),$$

where $K_{p}$ is the normalization factor, $N_{p}$ is the neighborhood of pixel p=(x,y). $\sigma_{s}$ and $\sigma_{r}$ control the spatial and range weights. The all-in-focus image FI is taken as the guidance image of the joint bilateral filter which smooths the depth map while preserving the edges. The anchored, rolling refinement continues until the depth map is visually indifferent between two iterations.

### 3.3. Guided Fusion

**Figure 3:**
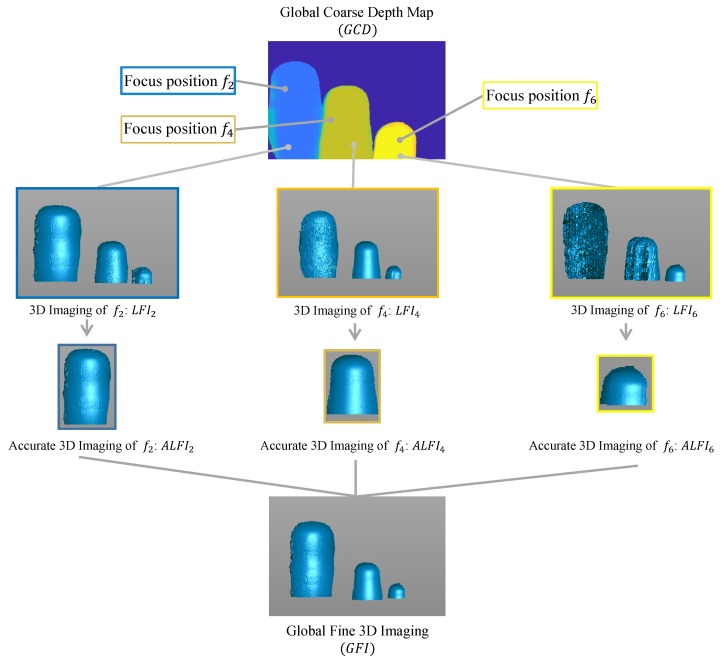
The guided fusion model. The global coarse depth map contains the corresponding focus position of each object. Therefore, we can extract the accurate region of each local fine 3D measurements under the guidance of the global coarse depth map to fuse into the global fine 3D measurement.

In this section, we derive a novel fusion model to generate the high-quality 3D measurement of the overall scene, which is shown in Figure 3. After the previously-described processes, we obtained multiple local 3D measurements of the scene. However, due to the working-range limitation of the traditional method, only objects in focus could be reconstructed well, while other, defocused objects could not. It is favorable to distinguish which objects are focused under the focal setting and extract the corresponding local 3D measurement. Fortunately, the global coarse depth map generated by the focus stacking technique could achieve this goal. The depth map stores the index of the image in the stack, which corresponds to the focus position of the image. Thus, we directly employ the depth map as the guidance map to extract the accurate part of each local 3D measurement. Regarding the depth map as a mask for each focus position, we define the mask function $\delta_{i}\left(x,y\right)$ for each focus position *i* as

(13) $$\begin{array}{c} \delta_{i}\left(x,y\right)=\left\{\begin{array}{cc} 1, & \mathrm{if}\;D(x,y)=i\\ 0, & \mathrm{if}\;D(x,y)\neq i \end{array}\right.. \end{array}$$

Then, the accurate part of each local 3D measurement ($ALFI_{i}$) is extracted under the guidance of each mask and they are fused into the global fine 3D measurement (GFI) by

(14) $$ALFI_{i}\left(x,y\right)=LFI_{i}\left(x,y\right)\delta_{i}\left(x,y\right)$$

(15) $$GFI\left(x,y\right)=\sum_{i=1}^{N}ALFI_{i}\left(x,y\right),$$

where *N* is the range of the index in the focal stack, and $LFI_{i}\left(x,y\right)$ is the 3D measurement of position (x,y) in the focus position *i*.

## 4. Experiments

In this section, we will present the experimental performance of our proposed method. In Section 4.1, we introduce the prototype system and the test scene. Section 4.2 evaluates the performance of our proposed method with the traditional method on real data. In Section 4.3, we analyze the reconstruction errors of our method with the traditional method. At last, Section 4.4 discusses the robustness of our method in the different scenes.

### 4.1. Setup

**Figure 4:**
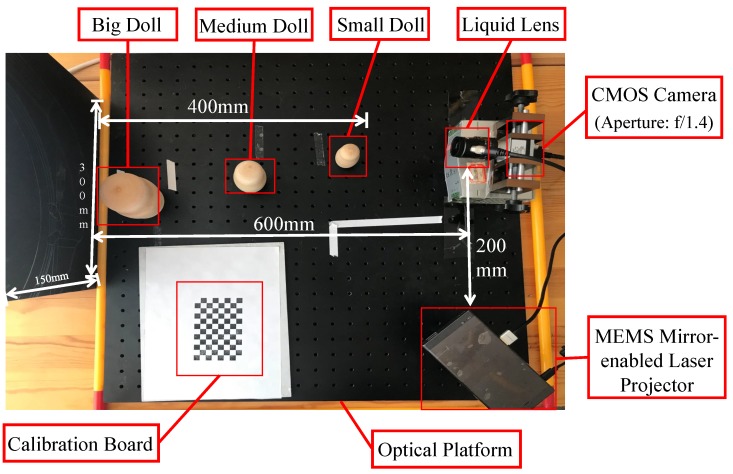
The prototype system and the test scene. The system includes a MEMS mirror-enabled laser projector, a CMOS camera equipped with a liquid lens, and a control platform for synchronization. Three different sizes of dolls are placed in front of the camera, from far to near. The specific scene is shown in the figure.

The prototype system and the test scene are shown in Figure 4. The system included a CMOS camera (model: Basler acA1920-155um, manufactured by the Basler AG, Ahrensburg, Germany) equipped with a 12 mm focal length lens (model: Computar m1214-mp2, manufactured by the Computar, Tokyo, Japan) and a liquid lens (model: Optotune EL-10-30-Ci, manufactured by the Optotune AG, Dietikon, Switzerland). A MEMS mirror-enabled laser projector (model: Sony MP-CL1A, manufactured by the Sony Corporation, Tokyo, Japan) was used for structured light projection. We adopted a high-precision electrical lens driver to control the liquid lens. The resolution of the camera was set to 1280×800 while the resolution of the projector was 1920×1200. The camera aperture was always set to the maximum of f/1.4 to maximize light throughput, which results in a small DOF effect. Thus, we can demonstrate the effectiveness of our proposed method to extend the DOF of the structured light 3D imaging system. In order to test the effect of our method in the depth range of 200–600 mm, we placed a small doll at the distance of roughly 200 mm, a medium doll at the distance of roughly 400 mm, and a big doll at the distance of roughly 600 mm. The depth variation of the scene was 400 mm, which was large compared to the relative distance between the scene and the baseline of the prototype system described before.

### 4.2. Overall Performance

In this experiment, we adopted six discrete focal distances for local 3D measurements based on the method in Section 3.1: $f_{1}$ = 100 mm, $f_{2}$ = 200 mm, $f_{3}$ = 300 mm, $f_{4}$ = 400 mm, $f_{5}$ = 500 mm, and $f_{6}$ = 600 mm; and their corresponding driver currents are 242.21 mA, 84.74 mA, 47.41 mA, 35.83 mA, 23.38 mA, and 16.73 mA. The union of the depth of fields of all the images in the focal stack covers the range of three dolls in the scene. After capturing the structured light patterns from $f_{1}$ to $f_{6}$, we need to calibrate the system with the corresponding focal setting. The liquid-lens-based imaging system allows us to accurately restore each focus position by setting driver current and performing precise calibration for 3D measurement [26].

**Figure 5:**
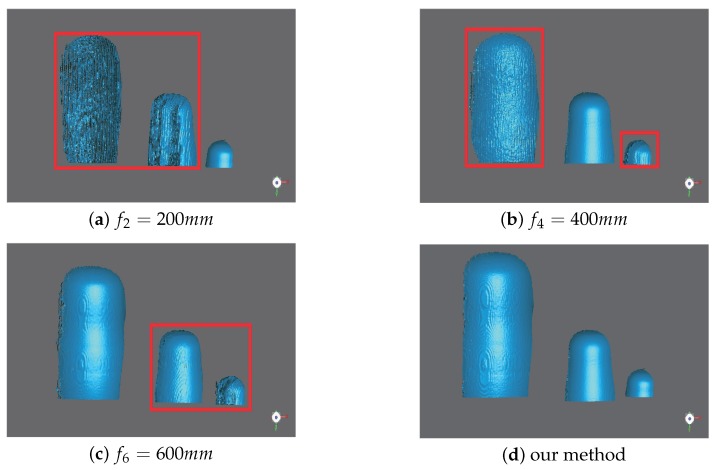
Comparison of measurement results. (**a**) The camera focal distance at $f_{2}=200$ mm; (**b**) the camera focal distance at $f_{4}=400$ mm; (**c**) the camera focal distance at $f_{6}=600$ mm; (**d**) our method.

To show the limitations of traditional methods and demonstrate the effectiveness of our method, we compare the results of local 3D measurements when *f* = 200 mm, 400 mm, and 600 mm with our proposed method in Figure 5. The parts within red rectangles correspond to objects which are not focused; thus, the measurement quality drops with the increasing amount of defocusing. Our method extracts the best quality 3D measurement from each local position and fuses them all into the global results; thus, achieving the best performance.

As shown in Figure 5, the surface of the reconstructed object is rough when the object is not focused, while the surface is smooth when the object is focused. Therefore, we can measure the smoothness of the surface to determine the quality of measurement by Geomagic studio. The "Reduce Noise" function provides three indicators for quantifying the smoothness of the surface of the object, which are maximum distance, average distance, and standard deviation. These three indicators are for the entire 3D reconstruction that includes both the in-focus region and the out-of-focus region, and they show information regarding the distance that points are moved during the smoothing process. Maximum distance indicates the greatest distance that any point is moved. Average distance indicates the average distance that all points are moved. Standard deviation indicates the variability of the distances. The comparison results are listed in Table 1. Our proposed method is better than the traditional method in terms of the maximum distance, average distance, and standard deviation, which fully demonstrates the superiority of our method.

### 4.3. Error Analysis

**Figure 6:**
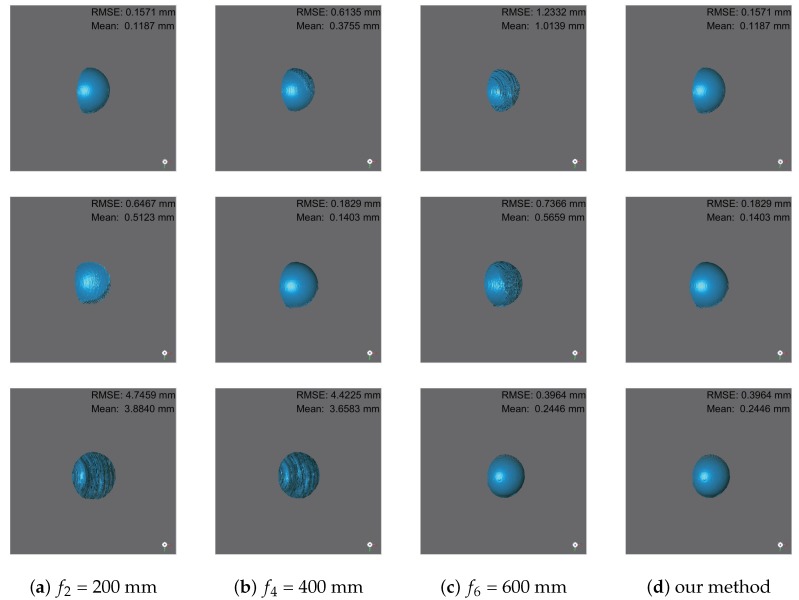
Comparison of reconstruction results. (**a**) $f_{2}$ = 200 mm; (**b**) $f_{4}$ = 400 mm; (**c**) $f_{6}$ = 600 mm; (**d**) our method. The first row represents the results of the small sphere (diameter = 30 mm at 200 mm) under different camera settings and methods. The second row represents the corresponding results of the medium sphere(diameter = 50 mm at 400 mm), and the last row represents the corresponding results of the big sphere(diameter = 70 mm at 600 mm). The reconstruction errors are shown in the upper right corner of each figure, which are RMSEs and mean errors.

We further quantitatively evaluated the reconstruction error by measuring the sphere in this section. The dolls were replaced with the same size spheres in each location, and then we fit the reconstruction data with an ideal spherical model. We set three focal distances of the camera $f_{2}$, $f_{4}$, and $f_{6}$ to ensure one sphere was focused in the image, respectively. The comparison diagram is shown in Figure 6 and the comparison results are listed in Table 2 and Table 3. In Figure 6, the first column shows the results of the first sphere (diameter = 30 mm at 200 mm), second sphere (diameter = 50 mm at 400 mm), and third sphere (diameter = 70 mm at 600 mm) when the camera set its focal distance at $f_{2}$. The second column shows the corresponding results of $f_{4}$, the third column shows the corresponding results of $f_{6}$, and the last column shows the corresponding results of our method. The RMSEs and the mean errors are their fitting errors with the corresponding ideal sphere, not for the entire scene. The average RMSEs of each focus setting were 1.7421 mm ($f_{2}$), 1.8474 mm ($f_{4}$), and 0.7887 mm ($f_{6}$), and the average mean errors of each focus setting were 1.4298 mm ($f_{2}$), 1.4666 mm ($f_{4}$), and 0.6081 mm ($f_{6}$). Our method extracts the accurate measurement of each local position into the final composited measurement. The RMSE and mean error of our method were 0.2455 mm and 0.1679 mm, which is an achievement of approximately a 70% decrease of each local measurement. The experiments quantitatively demonstrate that the reconstruction error increases as the objects move away from the camera’s focal plane, especially the region with large shape gradient. And our proposed method can effectively achieve the most accurate measurements with the guided fusion model.

### 4.4. Robustness Analysis

**Figure 7:**
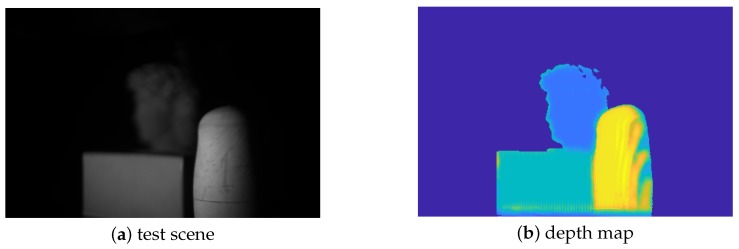
Photograph of the test scene and the global coarse depth map. (**a**) Test scene; (**b**) depth map.

**Figure 8:**
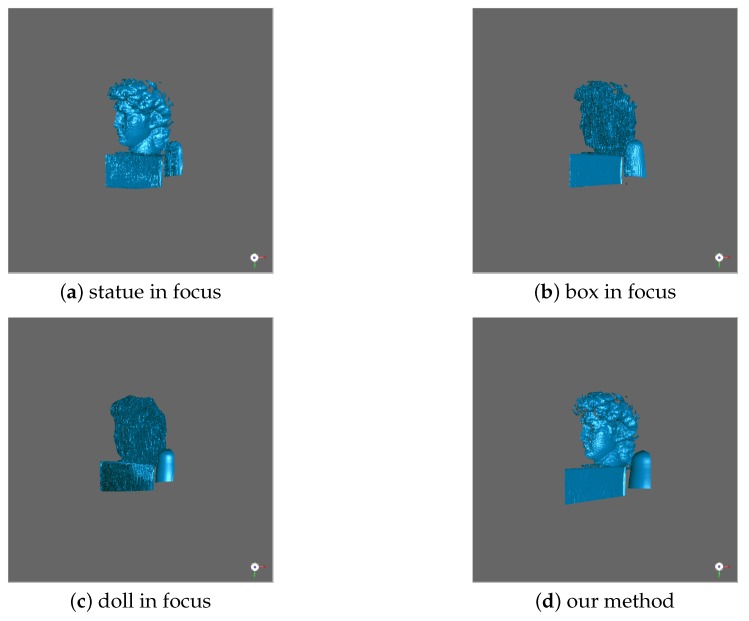
Comparison of measurement results. The scene includes three objects of different shapes and materials. (**a**) The statue in-focus; (**b**) the box in-focus; (**c**) the doll is in-focus; (**d**): our method.

In this section, we mainly analyze the robustness of our method in another two different scenes. The first scene includes various shapes and different surface reflectivity values, as shown in Figure 7a. In the process of capturing structured light patterns, we changed the focus position from near to far, and made each object in-focus at a certain focal setting. The “Global Depth Rough Estimation” module accurately extracted the focus position of each target from the focal stack and generated the corresponding depth map of the scene, which is shown in Figure 7b. We present the experiment results in Figure 8. Figure 8a shows the 3D measurement when the statue is in focus. Similarly, Figure 8b,c show the 3D measurements when the box and the doll are in focus, respectively. As discussed in Section 4.2, the best-quality 3D result is obtained when the object is in-focus and the measurement quality drops with the increasing amount of defocusing. The 3D measurement of our method is shown in Figure 8d, which perfectly preserved the shape of each target and achieved the best performance.

**Figure 9:**
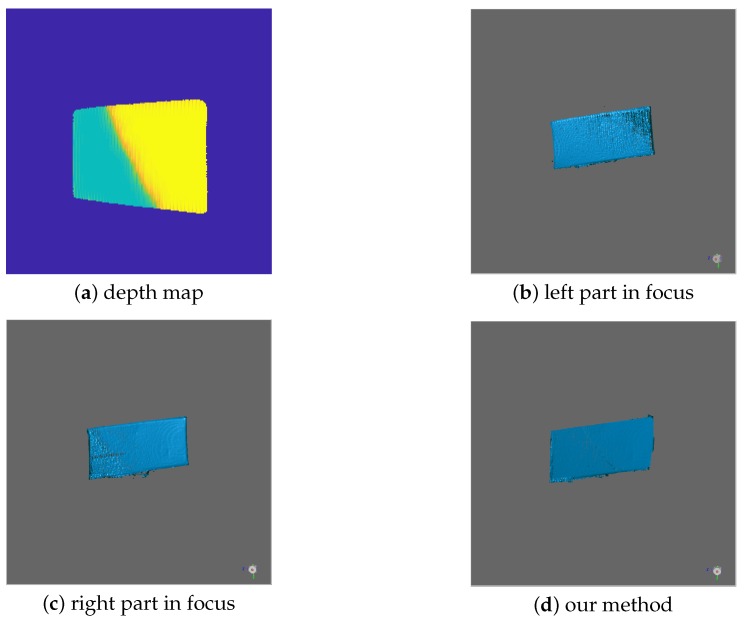
Comparison of measurement results. The scene includes one object having a large depth range. (**a**) The global coarse depth map; (**b**) the left part in-focus; (**c**) the right part in-focus; (**d**) our method.

The second scene consists of one object having a large depth range; thus, one focus position can only make its part in-focus. The depth map of the scene is shown in Figure 9a, which clearly indicates the focus position of each part. We present the experiment results in Figure 9b–d. Figure 9b shows the 3D measurement when the left part is focused, and Figure 9c shows the 3D measurement when the right part is focused. Our method extracts the best quality 3D measurements under the guidance of the depth map and fuses them into the final result, which is shown in Figure 9d. The experiments clearly demonstrate that our method can effectively work in a more complicated scene and achieve high-quality measurements.

## 5. Discussion

The proposed method achieved high-quality measurement of a large depth variation scene. Yet, it also makes some sacrifices. The major limitations associated with the proposed method are:

- *More time required*: since our method needs to capture images at different focus positions, the time taken by our method is more than that of the traditional methods.
- *High precision calibration required*: The proposed method has a strict requirement for calibration. Inaccurate calibration will introduce an artificial error, making measurement points of each focus position misaligned.

Despite these limitations, the values of the proposed methods are still substantial for high-accuracy and high-quality measurements of large-depth variation scenes. Besides, the time increase is adjustable according to the scene, and the calibration methods have been studied well in recent years.

## 6. Conclusions

In this paper, we proposed a novel method to extend the working range of structured light 3D imaging systems. We changed the traditional structured light 3D imaging system to the MEMS mirror-enabled laser projector and the liquid-lens-based imaging system. Based on the focus stacking technique, we developed a guided fusion model to generate a high-quality 3D measurement of the large depth variation scene. In experiments with the prototype system, we developed demonstrated that the proposed method could effectively work from a depth range of 200–600 mm with the measurement error of 0.08%.

## Figures and Tables

**Table 1:** Comparison of surface smoothness of reconstructed doll.

	Maximum Distance (mm)	Average Distance (mm)	Standard Deviation (mm)
$f_{2}$ = 200 mm	1.613432	0.121577	0.155088
$f_{4}$ = 400 mm	1.279587	0.134344	0.130969
$f_{6}$ = 600 mm	1.539015	0.325372	0.276541
Our method	0.919761	0.055475	0.047435

**Table 2:** Data comparison of fitting results in terms of RMSE (mm).

	Small Sphere	Medium Sphere	Big Sphere	Average Value
$f_{2}$ = 200 mm	0.1571	0.6467	4.7459	1.8499
$f_{4}$ = 400 mm	0.6135	0.1829	4.4225	1.7396
$f_{6}$ = 600 mm	1.2332	0.7366	0.3964	0.7887
our method	0.1571	0.1829	0.3964	0.2455

**Table 3:** Data comparison of fitting results in terms of mean error (mm).

	Small Sphere	Medium Sphere	Big Sphere	Average Value
$f_{2}$ = 200 mm	0.1187	0.5123	3.8840	1.5050
$f_{4}$ = 400 mm	0.3755	0.1403	3.6583	1.3914
$f_{6}$ = 600 mm	1.0139	0.5659	0.2446	0.6081
our method	0.1187	0.1403	0.2446	0.1679
